# The Nuclear Envelope Protein, LAP1B, Is a Novel Protein Phosphatase 1 Substrate

**DOI:** 10.1371/journal.pone.0076788

**Published:** 2013-10-07

**Authors:** Mariana Santos, Sandra Rebelo, Paula J. M. Van Kleeff, Connie E. Kim, William T. Dauer, Margarida Fardilha, Odete A. da Cruz e Silva, Edgar F. da Cruz e Silva

**Affiliations:** 1 Health Sciences Department, Centre for Cell Biology, Neuroscience Laboratory, University of Aveiro, Aveiro, Portugal; 2 Health Sciences Department, Centre for Cell Biology, Signal Transduction Laboratory, University of Aveiro, Aveiro, Portugal; 3 Departments of Neurology and Cell & Developmental Biology, University of Michigan Medical School, Ann Arbor, Michigan, United States of America; Institute of Enzymology of the Hungarian Academy of Science, Hungary

## Abstract

Protein phosphatase 1 (PP1) binding proteins are quintessential regulators, determining substrate specificity and defining subcellular localization and activity of the latter. Here, we describe a novel PP1 binding protein, the nuclear membrane protein lamina associated polypeptide 1B (LAP1B), which interacts with the DYT1 dystonia protein torsinA. The PP1 binding domain in LAP1B was here identified as the REVRF motif at amino acids 55-59. The LAP1B:PP1 complex can be immunoprecipitated from cells in culture and rat cortex and the complex was further validated by yeast co-transformations and blot overlay assays. PP1, which is enriched in the nucleus, binds to the N-terminal nuclear domain of LAP1B, as shown by immunocolocalization and domain specific binding studies. PP1 dephosphorylates LAP1B, confirming the physiological relevance of this interaction. These findings place PP1 at a key position to participate in the pathogenesis of DYT1 dystonia and related nuclear envelope-based diseases.

## Introduction

Reversible protein phosphorylation is a major mechanism controlling key intracellular events that are essential for cell health and viability [1-3]. Protein phosphatase 1 (PP1) is a ubiquitous serine/threonine phosphatase that is estimated to dephosphorylate about one third of all proteins in eukaryotic cells [4,5]. PP1 regulates a variety of cellular functions, such as glycogen metabolism, transcription, protein synthesis, cellular division and meiosis [4,6,7]. In mammalian cells, three genes encode the three PP1 isoforms: PP1alpha (PP1α), PP1Beta/Delta (PP1β/δ) and PP1gamma (PP1γ). Furthermore, the PP1γ gene undergoes alternative splicing to originate a ubiquitous PP1γ1 variant and a PP1γ2 variant that is enriched in testis [8,9]. PP1 isoforms are expressed in virtually all tissues but exhibit different expression levels depending on the tissue, and different subcellular distribution [9-11]. The versatility of PP1 is largely determined by the binding of its catalytic subunit to different specific regulatory subunits, that are responsible for the directed targeting of PP1 to a particular subcellular compartment and also determine its substrate specificity and activity [4,6,7,12]. More than 200 binding/regulatory subunits have been already described, making PP1 an essential protein in many distinct cellular processes [2,13]. Most PP1 binding proteins interact with the PP1 catalytic subunit through a conserved PP1 binding motif termed the RVxF motif, which has the consensus sequence [R/K] X_A(0-1)_ [V/I] X_B_ [F/W], where X_A_ is any amino acid and X_B_ is any amino acid except proline [14]. More recently, other consensus sequence have been proposed for the RVxF motif: [HKR]-[ACHKMNQRSTV]-V-[CHKNQRST]-[FW] [15] and [KRL] [KRSTAMVHNQ] [VI] {FIMYDP} [FW] [16]. The binding of PP1 to regulatory proteins through the RVxF motif does not cause major effects on the conformation and activity of PP1, but mediates the initial anchoring of regulatory subunits and thereby promotes the interaction at secondary binding sites [7,16,17]. Subsequently other PP1 binding motifs have also been reported, such as the apoptotic signature F-X-X-[KR]-X-[KR] [18], the SILK and the MyPhoNE motifs [16]. The existence of these conserved binding sites within regulatory subunits explains the ability of PP1 catalytic subunit to interact with numerous regulatory proteins and consequently the binding of most regulatory subunits is mutually exclusive [7]. Since the regulatory subunits control the specificity and the diversity of PP1 activity, the key to understanding PP1 function lies in studying these regulatory subunits and their cellular functions. Several yeast two-hybrid (YTH) screens of a human brain cDNA library were performed using the three PP1 isoforms (PP1α, PP1γ1 and PP1γ2) as baits. From each screen many positive clones were identified encoding several different proteins. Among them, were proteins already known as PP1 regulators and also novel putative PP1 regulators [19,20]. The majority of the novel putative PP1 regulators comprise at least one of the conserved PP1 binding motifs. One of the novel PP1 regulators isolated in the three independent YTH screens was torsinA interacting protein 1 (TOR1AIP1) or lamina associated polypeptide 1B (LAP1B). LAP1B belongs to a family of integral proteins of the inner nuclear membrane, named lamina associated polypeptide 1 (LAP1). Members of the LAP1 family (LAP1A, B and C) were initially identified using monoclonal antibodies generated against lamina-enriched fractions of rat liver nuclei [21]. The three LAP1 isoforms result from alternative splicing of the *TOR1AIP1* gene [22] and have been poorly studied. Moreover, the cDNA for LAP1B isoform was, to date, the only human isoform completely sequenced [23]. The function of LAP1B is poorly understood but it is known that it binds to lamins and chromosomes and that it is phosphorylated during interphase and mitosis [24]. As indicated by the nomenclature, LAP1 was found to interact with torsinA [25,26], the central protein of a neurologic disorder known as DYT1 dystonia [27]. However, the physiological relevance of this novel complex has not yet been determined. In the present study we validated the novel complex LAP1B:PP1 using several *in vitro* and *in vivo* techniques, namely a blot overlay assay, co-immunoprecipitation (co-IP) and yeast co-transformation. Furthermore, the PP1 binding motif responsible for the interaction was mapped. The functional relevance of this complex was pursued and we determined that LAP1B is dephosphorylated by PP1 *in vitro.*

## Materials and Methods

### Antibodies

The primary antibodies used were rabbit polyclonal LAP1 [25]; rabbit polyclonal CBC2C and CBC3C, that recognizes the C-terminal of PP1α and PP1γ, respectively [9]; rabbit polyclonal lamin B1 (Santa Cruz Biotechnology); 6×His-tag antibody (Novagen), that recognizes 6×His-tag-proteins; and Myc-tag antibody (Cell Signaling), that recognizes Myc-fusion proteins. The secondary antibodies used were anti-mouse and anti-rabbit horseradish peroxidase-linked antibodies (GE Healthcare) for ECL detection, and FITC-conjugated anti-mouse IgG (Molecular Probes) and Alexa 594-conjugated anti-rabbit IgG (Molecular Probes) for co-localization studies.

### Expression vectors and DNA constructs

For yeast co-transformation assays, PP1α, PP1γ1, PP1γ2 and the specific C-terminal of PP1γ2 (PP1γ2End) cDNAs were cloned into the pAS2-1 (Clontech), in frame with the GAL4-binding domain, as described in Esteves et al, 2012 [19,20]. pACT2-LAP1B (in frame with the GAL4 activation domain) was obtained from a human brain cDNA library (Clontech, HL4004AH) [19,20]. LAP1B binding motif (BM) deletion mutants comprising amino acids 1-209 (LAP1B-BM1), 61-508 (LAP1B-BM2), 1-508 (LAP1B-BM1/2) and 238-584 (LAP1B-BM3) were prepared by PCR amplification with appropriate primers (Table S1). The amplified fragments were subcloned into the *EcoRI/XhoI* restriction sites of the pACT2 vector (Clontech). The same methodology was used to clone additional LAP1B deletion mutants comprising amino acids 1-338 (LAP1B-BM1/2-TM), 1-369 (LAP1B-BM1/2+TM), 332-584 (LAP1B-BM3-TM) and 365-584 (LAP1B-BM3+TM) into the pET-28c vector (Novagen). Full-length LAP1B and the mutants 1-209 (LAP1B-BM1) and 61-508 (LAP1B-BM2) in the pACT2 vector were subcloned into the pET-28c vector. Full-length LAP1B and LAP1B-BM2 and LAP1B-BM3 in the pACT2 vector were also subcloned into the mammalian expression vector pCMV-Myc (Clontech) to obtain a Myc-fusion protein. Additionally a LAP1B (ΔA185) mutation was introduced into pET-LAP1B by site-directed mutagenesis using the primers described in Table S1. The constructs were all verified by DNA sequencing using an ABI PRISM 310 Genetic Analyzer (Applied Biosystems, Porto, Portugal).

### Yeast co-transformation analysis

Each of the bait plasmids (pAS2-1-PP1α, pAS2-1-PP1γ1, pAS2-1-PP1γ2 or pAS2-1-PP1γ2end) was co-transformed with one of the specific target proteins; pACT-LAP1B or its deletions mutants (pACT-BM1, pACT-BM2, pACT-BM1/2 and pACT-BM3), into the yeast strain AH109 by the lithium acetate method (according to the manufacturer’s instructions, Clontech). In parallel, co-transformations of the vectors pAS2-1 and pACT2 were performed as a negative control. The association of murine p53 (encoded by plasmid pVA3-1) and SV40 large T antigen (plasmid pTD1) was used as a positive control. The transformants were assayed for *HIS3*, *ADE2* and *MEL1* reporter genes. All positive clones were replated in SD/QDO medium containing X-α-Gal and incubated at 30°C for 2-4 days.

### Expression of recombinant proteins in *Escherichia coli*

*Escherichia coli* Rosetta cells (DE3) were transformed with one of the pET-LAP1B constructs and grown overnight in 5 mL of Luria-Bertani/Kanamycin medium at 37°C. Aliquots (500 µL) were transferred to 50 ml of Luria-Bertanini/Kanamycin until the OD_600_ was around 0.5-0.6. Expression was induced by adding 1 mM isopropyl-β-D-thiogalactopyranoside (IPTG) to the culture at 37°C for different periods of time (1, 3 and 5 hours) with shaking. Cells were recovered by centrifugation (4000 rpm for 10 min), resuspended in 500 µL of 1x PBS and lysed by sonication. Cells were then centrifuged (13200 rpm for 30 min), the supernatant was transferred to a new microtube (soluble fraction) and the pellet resuspended in 500 µL of boiling 1% SDS (insoluble fraction).

### Blot overlay assays

#### LAP1B deletion mutants

Bacterial extracts, prepared as described above, were separated on a 10% SDS-PAGE and the proteins were subsequently transferred to a nitrocellulose membrane. The membrane was overlaid with 1 µg/mL of purified recombinant PP1γ1 protein [28]. After washing to remove excess protein, bound PP1γ1 was detected by incubating the membrane with PP1γ antibody and developed by enhanced chemiluminescence (ECL, GE Healthcare).

#### LAP1B isoforms

LAP1B and LAP1B (ΔA185) proteins were generated by *in vitro* transcription/ translation (IVT) from pET-LAP1B and pET-LAP1B(ΔA185) expression vectors, respectively, using the TnT-coupled transcription/translation kit (Promega), according to the manufactures’ instructions. For the overlay assays, two samples of 250 ng of purified recombinant PP1γ1 protein [28] were separated on a 12% SDS-PAGE. Both proteins were transferred to a nitrocellulose membrane but one was overlaid with LAP1B-IVT, while the other was overlaid with LAP1B (ΔA185)-IVT. The bound proteins were detected by incubating the membrane with LAP1 antibody and developed by ECL.

### Cell culture and transfection

COS-7 (ATCC CRL-1651) and HEK293 (ATCC CRL-1573) cells were grown in Dulbecco’s modified Eagle’s medium (DMEM) supplemented with 10% Fetal Bovine Serum (FBS), 100 U/ml penicillin, 100 mg/ml streptomycin and 3.7 g/l NaHCO_3_ (Complete DMEM). SH-SY5Y cells (ATCC CRL-2266) were grown in Minimal Essential Medium (MEM) supplemented with F-12 Nutrient Mixture (Gibco, Invitrogen), 10% FBS (Gibco, Invitrogen), 1.5 mM L-glutamine, 100 U/mL penicillin and 100 mg/mL streptomycin (Gibco, Invitrogen). HeLa cells (ATTC CRM-CCL-2) were grown in Minimal Essential Medium (MEM) with Earle’s salts and GlutaMAX (MEM), supplemented with 10% fetal bovine serum, 1% MEM Non-Essential amino acids and 100 U/mL penicillin and 100 mg/mL streptomycin. All cultures were maintained at 37°C and 5% CO_2_. Transient transfections of COS-7 and HeLa cells were performed using LipofectAMINE 2000 (Invitrogen Life technologies). After 24 hours of transfection, cells were harvested for subsequent immunoprecipitation (IP) experiments or were fixed, using 4% paraformaldehyde, for immunocytochemical analysis.

### Brain dissection

Winstar rats (9-12 weeks) were obtained from Harlan Interfaune Ibérica, SL. All experimental procedures observed the European legislation for animal experimentation (2010/63/EU). No specific ethics approval under EU guidelines was required for this project, since the rats were only euthanized, by cervical stretching followed by decapitation, for brain removal. This is within the European law (Council Directive 86/609/EEC) and during this procedure we took all steps to ameliorate animal suffering and used the minimum number of animals possible. The procedures were approved and supervised by our Institutional Animal Care and Use Committee (IACUC): Comissão Responsável pela Experimentação e Bem-Estar Animal (CREBEA).

Briefly, animals were euthanized by cervical stretching followed by decapitation and the cortex was dissected out on ice. The tissue was then homogenized on ice, in non-denaturing lysis buffer (50 mM Tris-HCl pH 8.0, 120 mM NaCl, 4% CHAPS) containing protease inhibitors (1 mM PMSF, 10 mM Benzamidine, 2 µM Leupeptin, 1.5 µM Aprotinin, 5 µM Pepstatin A), with a Potter-Elvehjem tissue homogenizer with 10-15 pulses at 650-750 rpm [29]. Resulting tissue extracts were used for IP analysis using Dynabeads Protein G (Dynal, Invitrogen) as described below.

### Co-immunoprecipitation

COS-7 cells transfected with Myc-LAP1B were collected in lysis buffer (50 mM Tris-HCl pH 8, 120 mM NaCl, 4% CHAPS) containing protease inhibitors (1 mM PMSF, 10 mM Benzamidine, 2 µM Leupeptin, 1.5 µM Aprotinin, 5 µM Pepstatin A). Dynabeads Protein G (Dynal, Invitrogen) were washed in 3% BSA/1x PBS. Primary antibodies were cross-linked to Dynabeads according to the manufacter’s protocol. Cell lysates were precleared with 20 µL (0.6 mg) Dynabeads for 1 hour and then incubated with antibody-dynabeads for 2h at 4°C. The immunoprecipitates were washed in 1x PBS and proteins eluted by boiling in loading buffer before SDS-PAGE and immunoblotting analysis. For analysis of endogenous proteins, COS-7 cells and SH-SY5Y cells were collected in lysis buffer and immunoprecipitated as described above, but cell lysates were incubated with antibody-dynabeads overnight at 4°C. HEK293 immunoprecipitation was carried out as previously described [30]

### Immunocytochemistry

Once fixed as described above, HeLa cells were permeabilized with methanol for 2 min. Cells were first incubated with one of the primary antibodies (anti-PP1γ or PP1α) for 2 hours, followed by Alexa 594-conjugated secondary antibody. After washing with 1x PBS, cells were subsequently incubated with a second primary antibody (anti-myc) for 2 hours, followed by anti-mouse fluorescein isothiocyanate (FITC) conjugated secondary antibody. Preparations were washed with PBS, mounted using Vectashield mounting media with DAPI (Vector) and visualized using an LSM510-Meta confocal microscope (Zeiss) and a 63x/1.4 oil immersion objective. The argon laser lines of 405 nm, 488 nm, and a 561nm DPSS laser were used. Microphotographs were acquired in a sole section in the Z-axis (xy mode) and represent a mean of 16 scans. Profiles were acquired using the Zeiss LSM 510 4.0 software as previously described [29,31].

### *In vitro* dephosphorylation of LAP1B

SH-SY5Y cells were treated with 0.25 nM or 500 nM okadaic acid (OA) for 3 hours. Then, cells were collected in lysis buffer (50 mM Tris-HCl pH 8, 120 mM NaCl, 4% CHAPS, 250 mM EDTA, 1 mM sodium orthovanadate, 5 mM sodium fluoride) containing protease inhibitors (1 mM PMSF, 10 mM Benzamidine, 2 µM Leupeptin, 1.5 µM Aprotinin, 5 µM Pepstatin A) and immunoprecipitated with LAP1 antibody, as described above. Immunoprecipitates were incubated at 30°C for 1 hour with or without 100 ng of purified PP1γ1 protein in PP1 buffer (50 mM Tris-HCl pH 7.5, 0.1 mM EGTA, 1 mM MnCl_2_, 5 mM DTT). Samples were further analyzed by 7.5% SDS-PAGE followed by immunoblotting.

### SDS-PAGE and Immunoblotting

Samples were separated on SDS-PAGE and electrophoretically transferred onto nitrocellulose, followed by immunological detection with specific antibodies as indicated. Membranes were saturated in 5% non-fat dry milk in TBS-T for 3 hours and further incubated with primary antibodies. The incubations with the CBC2C, CBC3C and LAP1 antibodies were performed overnight. The incubations with the His-tag and Myc-tag antibodies were carried out for 2 hours. Detection was achieved using horseradish peroxidase-conjugated anti-rabbit or anti-mouse IgGs as secondary antibodies and proteins visualized by ECL (GE Healthcare).

## Results

### Identification of LAP1B as a novel putative PP1 regulatory protein

**Figure 1:**
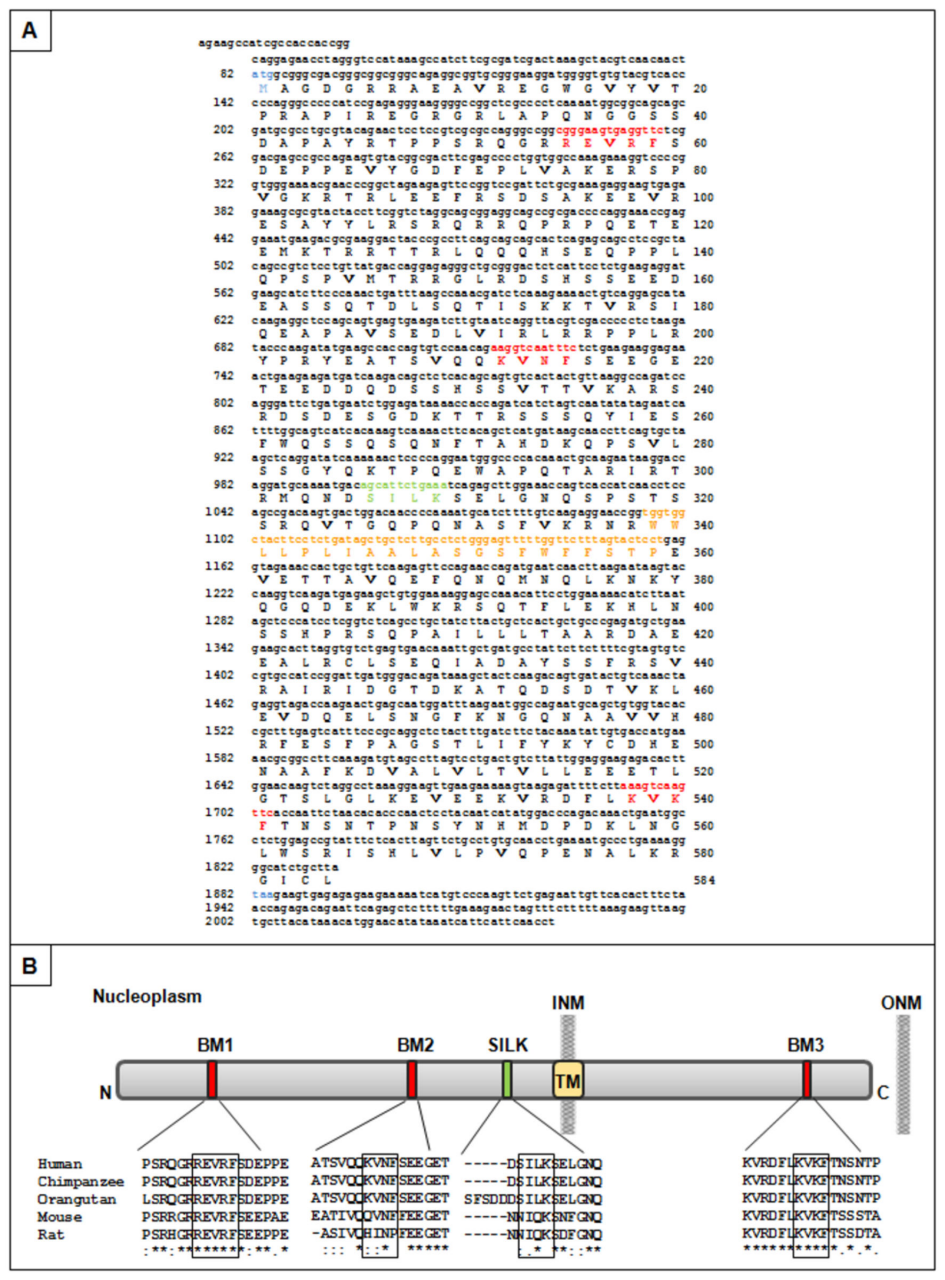
Human LAP1B sequence and domains. **A**- Nucleotide and corresponding amino acid sequence of human LAP1B encoded by clone 135. Stop and start codons are coloured blue. The three well conserved PP1 binding motifs (RVxF) are highlighted in red (positions 55-59, 212-215 and 538-541 in aa sequence) and a second generic PP1 binding motif (SILK) is coloured green (position 306-309 in aa sequence). The transmembrane domain is highlighted in orange (position 339-361 in aa sequence). **B**- Schematic illustration of LAP1B domains. Sequence of human LAP1B was aligned against others species using ClustalW algorithm. Sequence conservation is indicated by asterisks (identical sequences), colons (conserved substitutions) and periods (semi-conserved substitutions). BM1, BM2 and BM3, RVxF-like PP1 binding motif 1, 2 and 3, respectively; INM, inner nuclear membrane; ONM, Outer nuclear membrane; SILK, a second generic PP1 binding motif; TM, transmembrane domain.

In order to identify novel potential PP1 regulatory proteins, the three PP1 isoforms (PP1α, PP1γ1 and PP1γ2) were used as baits to screen a human brain cDNA library by the YTH system. From these screens 298, 241 and 228 positive clones were identified using PP1α, PP1γ1 and PP1γ2 as baits, respectively [19,20]. Detailed analysis revealed 14, 4 and 2 positive clones, encoding a potentially novel PP1 regulatory protein - LAP1B, for the PP1α, PP1γ1 and PP1γ2 screens, respectively (Table S2) [32]. Sequencing of the identified clones determined that all correspond to the LAP1B variant 1, recently reported in the GenBank database (NM_001267578.1). The variant 1 differs from the variant 2 (NM_015602) only by a CAG insertion, which results in an additional alanine in the coding sequence, otherwise the sequence is identical to the first human LAP1B sequence reported in 2002 [23]. Moreover, others reports showed that *TOR1AIP1* gene possesses a 3’ tandem splice site, TAGCAG, at the exon 3 boundary, which results in an amino acid insertion or deletion in the encoded protein [33,34]. From all LAP1B clones obtained we selected a full-length clone (clone 135; Table S2) for further studies. The latter comprises a short 5’ untranslated region followed by the ATG start codon, an open reading frame of 2000 nucleotides, that encodes 584 amino acids, followed by a stop codon and a short 3’ untranslated region (Figure 1A). LAP1B was previously described as a type 2 integral membrane protein, with an N-terminal nucleoplasmic domain, one predicted transmembrane domain (TM) and a C-terminal lumenal domain [23]. Our *in silico* analysis also revealed that LAP1B has three conserved PP1 binding motifs (BM): REVRF (amino acids 55-59), KVNF (amino acids 212-215) and KVKF (amino acids 538-541), that were called BM1, BM2 and BM3, respectively. BM1 and BM2 are localized in the nucleoplasm, while BM3 is localized in the lumen of the perinuclear space. Additionally, a second generic PP1 binding motif termed SILK (amino acids 306-309) was also identified (Figure 1A). Further *in silico* characterization of these four potential PP1 binding motifs (BM1, BM2, BM3 and SILK) was achieved using the ClustalW algorithm, which allowed for the determination of the homology between species (Figure 1B). Interestingly, the BM1 and BM3 are totally conserved motifs among the species analyzed (Human, Chimpanzee, Orangutan, Mouse, Rat). The other binding motifs (BM2 and SILK) are also completely conserved between Human, Chimpanzee and Orangutan but do deviate markedly in mouse and rat; with two residues common to all species in the SILK domain and only one residue is common to all species for the BM2 domain (Figure 1B). Subsequent to the *in silico* LAP1B characterization, the novel LAP1B:PP1 complex was validated *in vitro* and *in vivo*.

### LAP1B and PP1 interact *in vitro*

**Figure 2:**
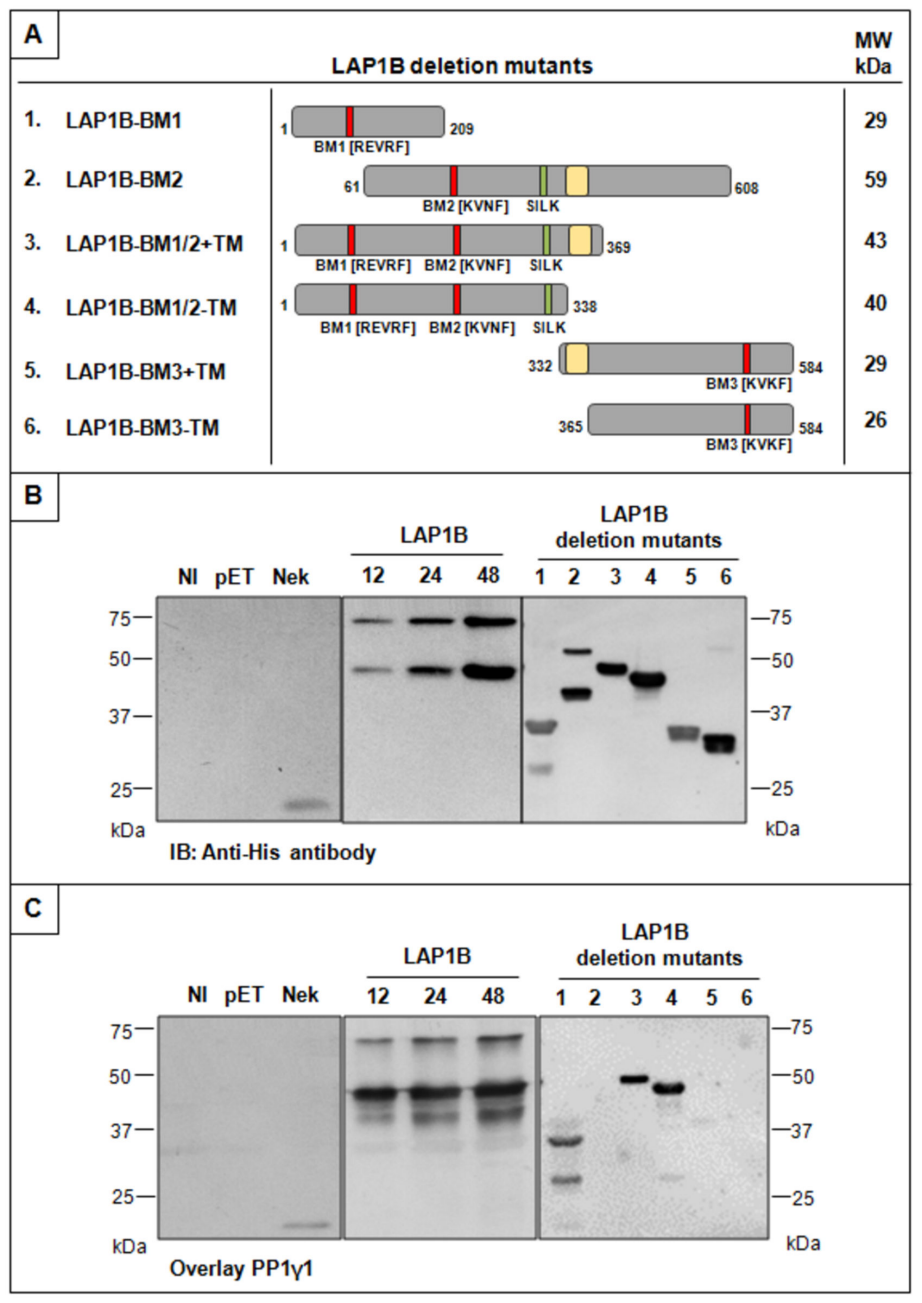
Blot overlay assay with PP1γ1. **Immunoblotting analysis using a His-tag antibody is also shown**. A-Schematic representation of LAP1B deletion mutants cloned into the pET-28c vector. The expected molecular weight (MW) of each construct is indicated. The red boxes represent the RVxF motifs, the green boxes correspond to the SILK motif, and the yellow boxes represent the transmembrane domain. B- Blot overlay assay of full-length LAP1B. Increasing amounts of recombinant full-length LAP1B (12, 24 and 48 µL) were loaded on each well as indicated. C- Blot overlay assay of LAP1B deletion mutants. Deletion mutants: 1, LAP1B–BM1; 2, LAP1B–BM2; 3, LAP1B–BM1/2+TM; 4, LAP1B–BM1/2-TM; 5, LAP1B-BM3+TM; 6, LAP1B-BM3-TM. Non-induced (NI) and pET-28c vector without an insert (pET) were used as negative controls and Nek2A (Nek) as positive control. Bacterial cultures were collected 3 hours after IPTG (1mM) induction at 37°C.

The *in vitro* validation of this novel complex as well as the identification of the binding motif responsible for the interaction was accomplished using a blot overlay assay. Several deletion mutants were prepared as described above, each comprising at least one of the PP1 binding motifs as represented in Figure 2A. The importance of the TM was also addressed by evaluating the binding in the presence or absence of the latter (Figure 2A). Thus, both full-length LAP1B and the different deletion mutants were expressed in bacteria as 6×His-tag fusion proteins by adding 1 mM IPTG to the growing cultures at 37°C. All recombinant proteins (full-length and mutants) were efficiently expressed in bacteria, including the deletion mutants with or without the TM domain (data not shown). The proteins thus expressed were used to carry out the blot overlay assay. Briefly, the different recombinant proteins were separated by SDS-PAGE and electrotransferred onto nitrocellulose membranes. Recombinant protein detection was achieved with the His-tag antibody (Figure 2B) and for the overlay assay the purified PP1γ1 protein was applied (Figure 2C). The negative controls used included the pET-28c vector without an insert or with a non-induced insert. As a positive control the pET28c-Nek2A was used, given that Nek2A is a well known protein that strongly interacts with PP1 [35]. The immunoblotting using His-tag antibody (Figure 2B) of both full-length and deletion mutants revealed that all the expressed proteins have the expected molecular weight as indicated in Figure 2A. Additional bands were sometimes detected for LAP1B, LAP1B-BM1 and LAP1B-BM2, these could possibly correspond to proteolytic fragments (Figure 2B). The overlay assays revealed increased PP1γ1 binding concomitant with increasing amounts of recombinant full-length LAP1B (Figure 2C). Additionally, we also demonstrated that PP1γ1 binds to the deletion mutant that comprises the residues 1-209 encompassing the BM1, and also to the construct that comprises both BM1 and BM2 with or without the TM (Figure 2C). However, PP1γ1 does not bind to the recombinant protein comprising only the BM2 or the BM3 (Figure 2C). Thus, using *in vitro* techniques we established that LAP1B binds to PP1γ1 through the BM1 (REVRF).

### The novel complex LAP1B:PP1 is also formed *in vivo*

**Figure 3:**
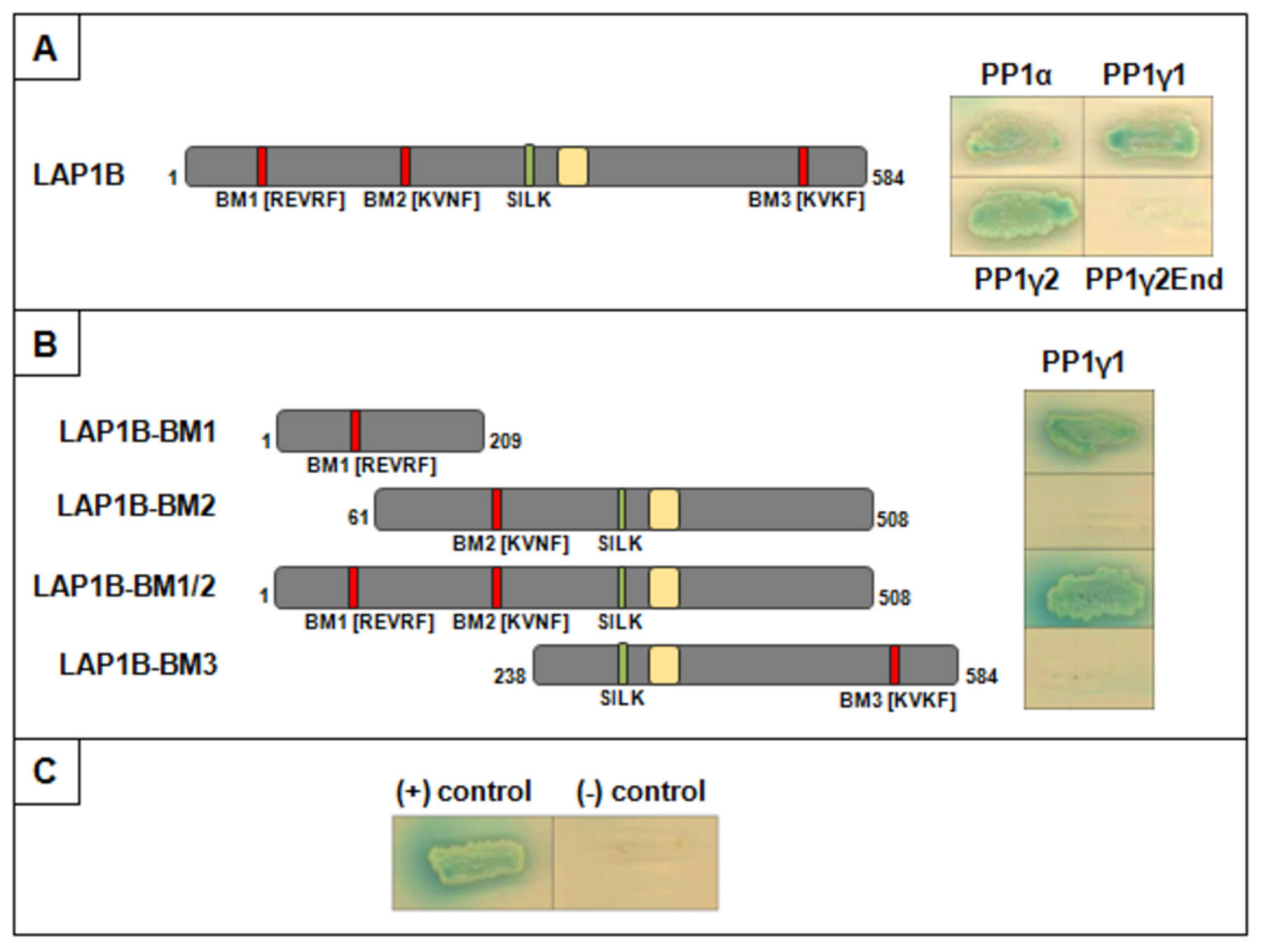
Yeast co-transformation assay in SD/QDO/X-α-gal medium. Full-length LAP1B and its deletion mutants cloned in the pACT2 vector are represented. The red boxes represent the RVxF motifs present in the constructs, the green boxes correspond to the SILK motif, and the yellow boxes represent the transmembrane domain. A- Positive interactions were observed between LAP1B and PP1α, PP1γ1 and PP1γ2, but not with the C-terminus of PP1γ2 (PP1γ2End). B- PP1γ1 interacted with LAP1B-BM1 and BM1/2 but not with LAP1B-BM2 and BM3. C- Association of murine p53 and SV40 large T antigen was used as positive control (+) and co-transformation of pAS2-1 and pACT2 vectors as negative control (-).

Having shown that the LAP1B:PP1 complex is formed *in vitro* the ability of the same to be formed *in vivo* was addressed. Yeast co-transformations were carried out in order to validate the novel complex formation and to simultaneously confirm relevant domains in LAP1B for the interaction, as well as to test different PP1 isoforms. Firstly, co-transformations were carried out with full-length LAP1B and different PP1 isoforms (α, γ1 or γ2), the assay took advantage of α-Galactosidase activity in the presence of the blue chromogenic substrate X-α-gal, producing a blue colour in the event of an interaction. The results (Figure 3) clearly show that LAP1B interacts with all PP1 isoforms tested (PP1α, PP1γ1 and PP1γ2; Figure 3A) but does not interact with the C-terminal portion of PP1γ2 isoform (PP1γ2end; Figure 3A). Subsequently, the relevant domain in LAP1B for the interaction was again tested using the *in vivo* conditions. These LAP1B deletion mutants, previously produced, were co-transformed in yeast with the PP1γ1 isoform and the results are presented in Figure 3B. Unequivocally, the results were positive for the deletion mutant that comprises the residues 1-209 including the BM1 (Figure 3B) and also with the construct that comprises both BM1 and BM2 (BM1/2). In sharp contrast the results were negative with the LAP1B-BM2 and LAP1B-BM3 (Figure 3B). The positive and negative controls are presented in Figure 3C. These results show that it is the BM1 (REVRF) that mediates the interaction between LAP1B and PP1.

**Figure 4:**
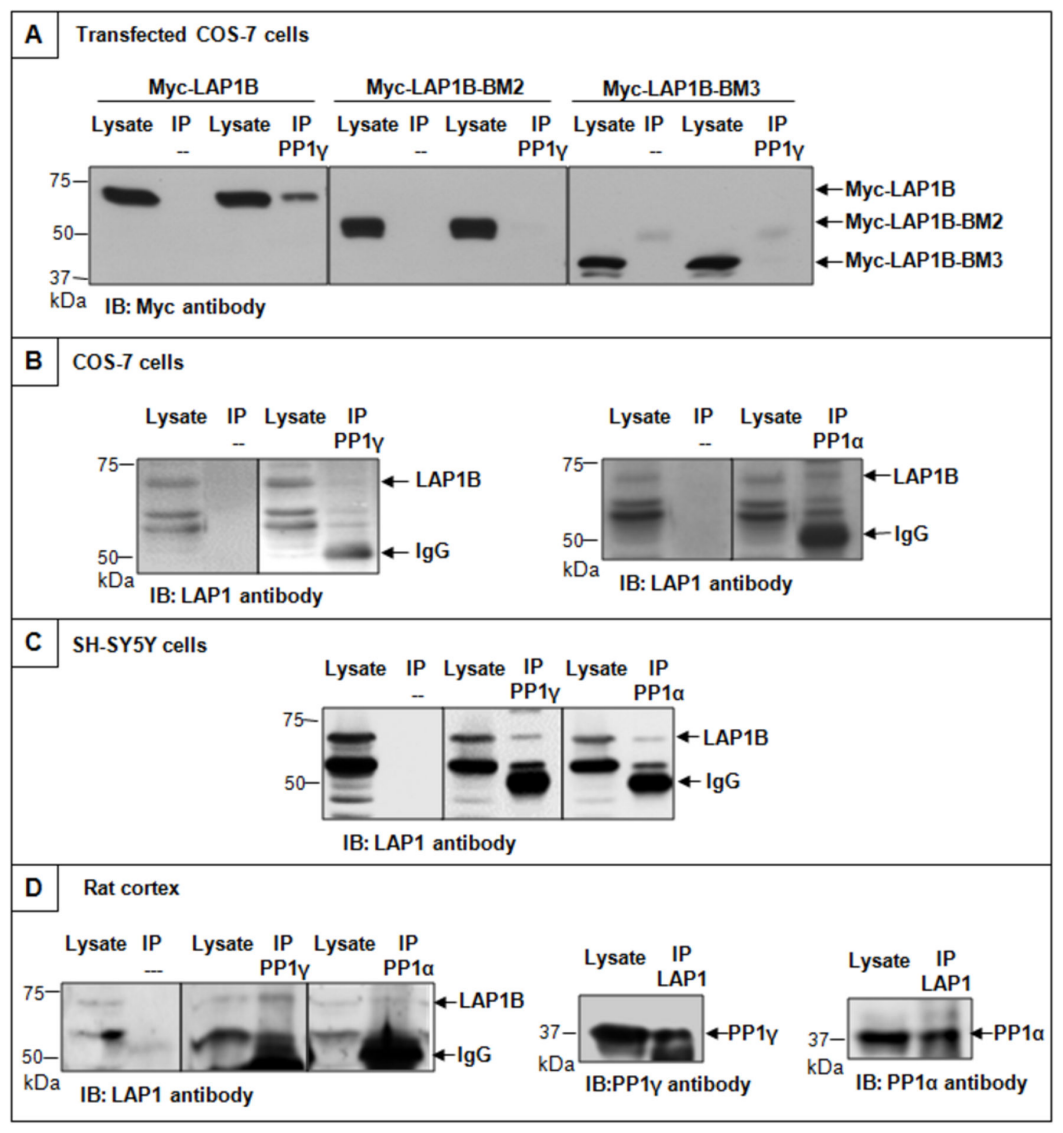
Co-immunoprecipitation of the PP1:LAP1B complex in COS-7 cells, SH-SY5Y cells and rat cortex. A- COS-7 cells were transfected with Myc-LAP1B, Myc-LAP1B-BM2 or Myc-LAP1B-BM3 and immunoprecipitated with PP1γ bound to protein G- DynaBeads. B- Non-transfected COS-7 cells were immunoprecipitated with PP1γ or PP1α antibodies bound to protein G- Dynabeads. C-SH-SY5Y cells were immunoprecipitated with PP1γ or PP1α antibodies bound to protein G- Dynabeads. D- Rat cortex extracts were immunoprecipitated with PP1γ, PP1α or LAP1 antibodies bound to protein G- Dynabeads. The negative controls were performed by incubating cell extracts with beads. IP, immunoprecipitation. IB, immunoblotting.

Additionally, the *in vivo* occurrence of the complex was further investigated and distinct models were used, namely a non-neuronal cell line (COS-7), a neuronal-like cell line (SH-SY5Y) and rat brain. Thus, co-IPs using the specific antibodies against PP1α, PP1γ and LAP1B were carried out. Firstly, COS-7 cells were transfected with Myc-LAP1B and Myc-LAP1B deletion mutants (LAP1B-BM2 and LAP1B-BM3) and further immunoprecipitated using the PP1γ antibody (Figure 4A). The Myc-LAP1B deletion mutants are similar to those tested in yeast co-transformation assays. Our results showed that PP1γ binds only to the full-length LAP1B but not to LAP1B deletion mutants (LAP1B-BM2 and LAP1B-BM3). Once more our results clearly showed that PP1γ only binds LAP1B when the BM1 is present. Non-transfected COS-7 (Figure 4B) and SH-SY5Y (Figure 4C) cellular extracts were also immunoprecipitated with PP1α and PP1γ antibodies. Rat cortical extracts were also immunoprecipitated with the same antibodies as well as with the LAP1 antibody and the results are presented in Figure 4D. Endogenous LAP1B was also detected after IP with PP1α and PP1γ in COS-7 cells (Figure 4B) and SH-SY5Y cells (Figure 4C). Finally, using rat cortical brain extracts we confirmed that the LAP1B:PP1 complex is also formed in brain, since we detected LAP1B after IP with PP1α or γ antibodies. Further, when we immunoprecipitated with LAP1 antibody we could also detect both PP1 isoforms (Figure 4D). The complex was also detected in other brain regions, namely rat striatum and cerebellum (data not shown) were PP1 is highly abundant.

### Both LAP1B isoforms bind to PP1

**Figure 5:**
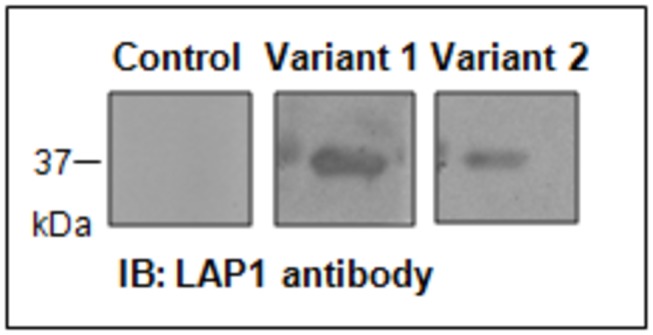
Blot overlay assay of LAP1B variants. Two samples of purified recombinant PP1γ protein were separated by SDS-PAGE and the resulting blot was overlaid with LAP1B-IVT (1) or LAP1B (ΔA185)-IVT (2). IB, immunoblotting.

Thus far we have unequivocally shown that LAP1B is a novel PP1 regulatory protein and that the complex formed by the two proteins can occur both in vitro and *in vivo*. Given the high degree of similarity between both LAP1B variants and the fact that both comprise the BM1 (REVRF) responsible for the interaction, it is reasonable to deduce that both variants interact with PP1. In order to confirm this hypothesis we generated, by site directed mutagenesis, the LAP1B (ΔA185) construct that corresponds to LAP1B variant 2 reported in GenBank. The interaction of both LAP1B isoforms with PP1 was indeed confirmed by *in vitro* overlay assay. Briefly, 250 ng of recombinant purified PP1γ1 protein were separated by SDS-PAGE and electrotransferred to a nitrocelulose membrane that was subsequently overlaid with each of the LAP1B variants. As expected both LAP1B variants are able to interact with PP1γ1 since we can detect a band of approximately 37 kDa (Figure 5) that corresponds to the molecular weight of PP1γ1.

### Localization of the LAP1B:PP1 complex

**Figure 6:**
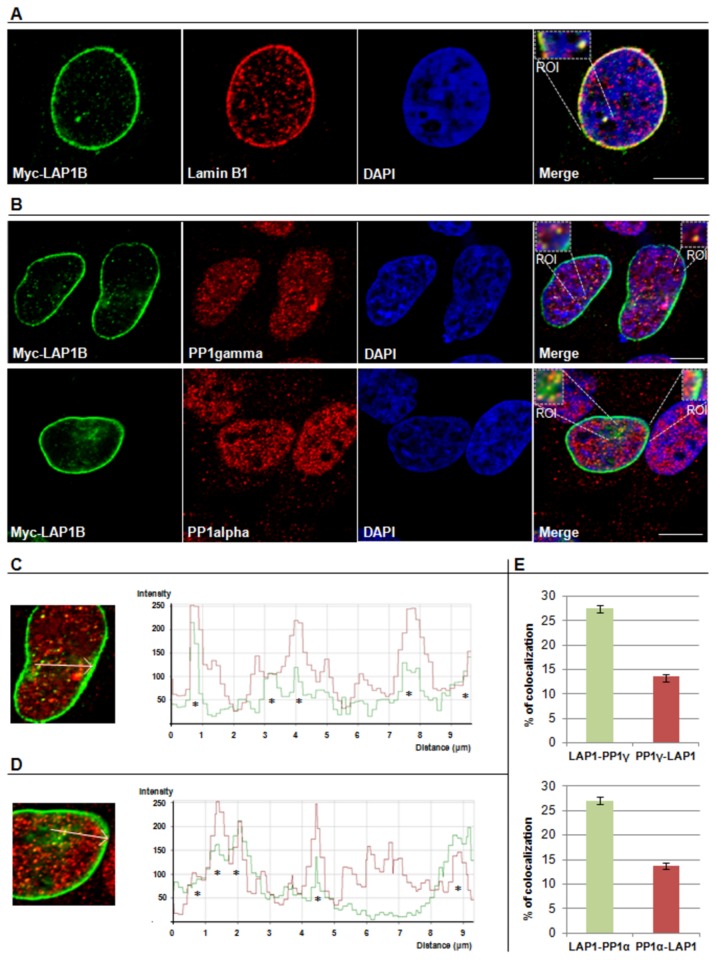
Subcellular distribution of the LAP1B:PP1 complex in HeLa cells. HeLa cells were transfected with Myc-LAP1B and then processed for immunocytochemistry using specific antibodies to Myc-tag and endogenous lamin B1 and PP1γ and α isoforms. **A**- Immunolocalization of both myc-LAP1B and lamin B1. **B**- Immunolocalization of myc-LAP1B and PP1γ and α isoforms. The presence of the complexes is evidenced by the ROI (region of interest). **C, D**- Confocal profiles representing the green fluorescence intensity (FITC-conjugated secondary antibody labelling Myc-LAP1B) and the red fluorescence intensity (Alexa Fluor 594- conjugated secondary antibody labelling PP1γ [C] or PP1α [D]) in a specific distance (arrow); asterisks denote co-localizing points. **E**- Quantification of % of co-localization between LAP1B and PP1 isoforms. Values are mean ± SEM, n= 75 cells (for PP1γ) and 55 cells (for PP1α). Photographs were acquired using a LSM 510-Meta confocal microscope. Bars, 10 µm.

Given the confirmation that this novel complex is formed *in vivo*, it is evident that these two proteins are functionally related and therefore it becomes important to describe their localization. Co-localization of both proteins indicated that the complex exists *in vivo* and has physiological relevance. HeLa cells were transiently transfected with Myc-LAP1B and subjected to immunocytochemistry using a Myc-tag antibody. The Myc-LAP1B was mainly found in the nuclear envelope and also in the nucleus where it co-localizes with lamin B1 (Figure 6A). Endogenous PP1γ and PP1α were detected using specific antibodies. All proteins have the expected subcellular distribution; each has been previously described individually [10,21,23]. The PP1γ was found predominantly in the nucleus, including the nucleolus, and throughout the cytoplasm (Figure 6B). The PP1α was also found predominantly in nucleus, excluding the nucleolus, and throughout the cytoplasm (Figure 6B). Despite the quite different subcellular distribution of LAP1B and PP1 isoforms it is evident that they co-localize at specific points within the nucleus and very near to the nuclear envelope, as observed by the yellow color indicated in the ROIs (Figure 6B). The co-localizing points were confirmed by confocal profiling (denoted with *), where we demonstrated that the fluorescence intensity of the two channels correlated, particularly in those points, through the white line represented in the image (Figure 6C and D). The co-localization quantitative analysis was performed (Figure 6E) using a specific co-localization software (Zeiss LSM 510 4.0 software) as previously described [29,31]. Essentially, LAP1B co-localizes similarly with both PP1 isoforms (27.6 ± 0.65% and 27.1 ± 0.8% with PP1γ and PP1α, respectively). The percentage of PP1γ and PP1α that co-localize with LAP1B is lower (13.5 ± 0.48 and 13.8 ± 0.8, respectively).

### PP1 specifically binds to LAP1B

**Figure 7:**
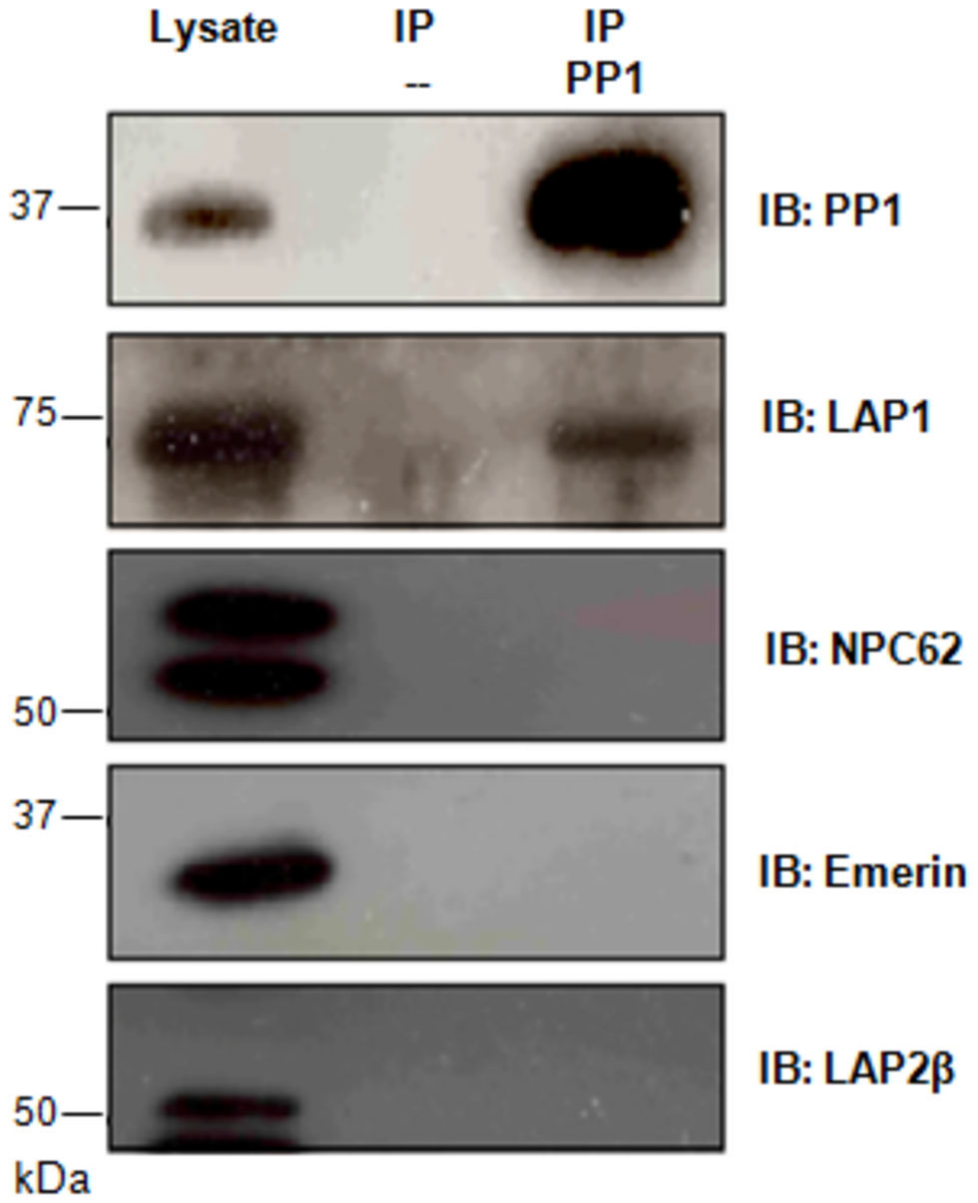
Co-immunoprecipitation of PP1 binding proteins at the nuclear envelope. HEK293 cells were immunoprecipitated with PP1 antibody bound to protein A- sepharose beads. The negative controls were performed by incubating cell extracts with beads. IP, immunoprecipitation. IB, immunoblotting.

Many nuclear membrane proteins are primarily or ultimately linked to each other via direct or indirect interactions involving the nuclear lamina. To test the specificity of the LAP1B:PP1 complex we tested whether other inner nuclear membrane proteins (NPC62, emerin and LAP2β) also bound to PP1. Co-IPs were performed using PP1 antibody and the potential binding partners were further screened using specific antibodies (Figure 7). The results presented, clearly show that PP1 only binds to LAP1 and not to the other proteins tested (NPC62, emerin and LAP2β). These results strengthen our hypothesis that LAP1 and PP1 are functionally associated, since they bind specifically in regions near the nuclear envelope (Figure 6).

### LAP1B is a novel substrate for PP1

**Figure 8:**
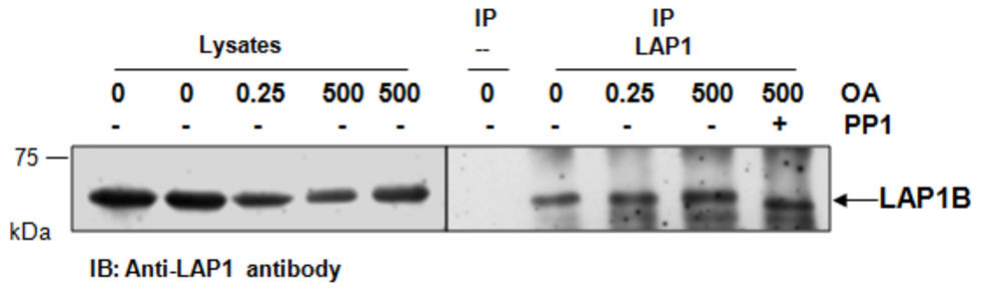
*In vitro* dephosphorylation of LAP1B. SH-SY5Y cells were incubated with 0, 0.25 or 500 nM okadaic acid (OA) for 3 hours and immunoprecipitated with LAP1 antibody. Immunoprecipitates were incubated at 30°C for 1 hour with or without 100 ng of PP1γ1 protein. The negative controls were performed by incubation of cells extracts with beads. In the left panel are presented the cell lysates correspondent of each condition immunoprecipitated (right panel). IP, immunoprecipitation. IB, immunoblotting.

It is well established that PP1 versatility is largely determined by its regulatory proteins. The latter define subcellular targeting, substrate specificity and even the activity PP1. Having determined that LAP1B is a novel PP1 regulatory protein, since the complex is formed both *in vitro* and *in vivo* and the two proteins co-localize in human cell lines, it is reasonable to deduce that the two proteins are functionally associated. We went on to test if LAP1B is a substrate for PP1, given that LAP1B can be phosphorylated at several residues [36-38]. SH-SY5Y cells were incubated with two different concentrations of OA (a protein phosphatase inhibitor), followed by IP with LAP1 antibody and further incubation with recombinant purified PP1γ1 protein (Figure 8). Analysis of Figure 8 showed that when we inhibit PP2A and PP1 (500 nM OA) a slight decrease in the migration of LAP1B is detected, consistent with an increase in its protein phosphorylation level, this is not evident when only PP2A is inhibited (0.25 nM OA) (Figure 8, lysates). In contrast, when we immunoprecipitate LAP1B from cells treated with 500 nM OA and incubated the resulting immunoprecipitates with 100 ng of purified PP1γ1 protein (Figure 8, IP) a clear shift in the opposite direction is observed, indicating an *in vitro* dephosphorylation by PP1γ1.

## Discussion

PP1 regulates numerous cellular functions by binding to different regulatory subunits, which are, in turn, responsible for the targeting of PP1 to a particular subcellular compartment, but also determine substrate specificity and activity [4,6,7,12]. In this study we show that LAP1B is a novel PP1 regulatory protein. This LAP1B interaction was originally identified in YTH screens of a human brain cDNA library using three PP1 isoforms (α, γ1 and γ2) as baits [19,20]. Moreover, LAP1B variant 1, from all these screens, was isolated and fully sequenced [32]. In each case the variant obtained was the one recently reported in the GenBank database (NM_001267578) and differs from the LAP1B variant 2 (NM_015602) by only a 3 nucleotide (CAG) insertion, which results in the introduction of a single alanine residue at position 185 of the coding sequence. Typically such variants are generated by alternative splicing [28,33] and this has been shown to be so for the *TOR1AIP1* gene. Despite this subtle difference, both LAP1B variants have the same conserved PP1 binding domains and bind to PP1 (Figure 5). Consistent with the YTH results the data here presented, for the yeast co-transformation assays, confirmed that LAP1B is able to bind to all the PP1 isoforms tested (α, γ1 and γ2), but not to the C-terminus of PP1γ2 isoform (Figure 3). Thus, this novel LAP1B:PP1 complex was validated not only *in vivo* but also *in vitro* by a blot overlay assay (Figure 2). LAP1 is expressed in both non-neuronal and neuronal tissues [30], as are PP1α and PP1γ1; even though the latter has higher expression levels in brain [9]. Thus, we performed co-IP assays in COS-7 cells (non-neuronal cell line), SH-SY5Y cells (neuronal-like cell line) and rat cortex (Figure 4). Moreover, the complex LAP1B:PP1 was also found in rat hippocampus and striatum, regions where PP1α and PP1γ are particularly enriched (data not shown)

LAP1B is not a well characterized protein and its main cellular function remains to be defined. However, it is an integral membrane protein of the inner nuclear membrane that interacts with lamins and chromosomes and this is functionally relevant for the maintenance of the nuclear architecture during interphase and mitosis [24]. Recently, it was demonstrated that LAP1 recruits torsinA to the nuclear envelope [25], although the latter is normally located at the endoplasmic reticulum. In contrast, a torsinA mutant form (ΔE302/303-torsinA), which is present in DYT1 dystonia patients, is primarily relocated to the nuclear envelope [39,40]. Protein: protein interactions can be regulated by protein phosphorylation and consequently by protein kinases and phosphatases. It is therefore highly relevant that LAP1B should bind to PP1. Thus, several LAP1B deletion mutants were generated to determine the binding region responsible for the binding to PP1. *In silico* analysis revealed that LAP1B has three potential PP1 binding RVxF motifs: REVRF (amino acids 55-59) and KVNF (amino acids 212-215) located in the nucleoplasm and KVKF (amino acids 538-541) located in the lumen of the perinuclear space. An additional generic PP1 binding motif termed SILK (Figure 1) is also present in the nucleoplasmic domain. We defined that LAP1B:PP1 interaction occurs primarily through a region that comprises the REVRF domains (BM1). These results (Figures 2C, 3B, 4A) support an interaction, which in topological terms provides physiological relevance, given that the BM1 is located in the nucleoplasmic portion of LAP1B and PP1 is particularly abundant in the nucleus. The BM2 and the SILK motif are also located in the nucleoplasm but our results clearly showed that these motifs do not mediate the interaction between LAP1B and PP1. BM3 is located in the perinuclear space making this an unlikely domain for the association of both proteins. Moreover, BM1 is conserved among different species (Figure 1B) while BM2 and SILK are not. Another remarkable aspect is that RVxF motif is often N-terminal flanked by basic residues and C-terminal flanked by acidic residues and this affects the binding affinity for the RVxF motif [14]. Concordantly, BM1 is preceded by basic residues (arginine) and followed by acidic residues (aspartic and glutamic acid) (Figure 1B). Further, the SILK motif is always N-terminal to the RVxF motif [16], but this is not the case with LAP1B where in fact it is C-terminal to the BM1, reinforcing that the SILK motif is not important for the interaction.

PP1 regulatory proteins can be substrates that directly associate with the PP1 catalytic subunit, but can also be substrate specifiers and/or targeting proteins. Having established that LAP1B is a novel PP1 regulatory protein and that the BM1 is responsible for the interaction, the physiological significance of this complex was addressed. Immunolocatization of LAP1B and PP1 (for both PP1α and PP1γ isoforms) showed that they co-localize near the nuclear envelope (Figure 6). These results are in agreement with PP1 interacting with LAP1B through its nucleoplasmic domain, as discussed above. Furthermore, LAP1B is well described to interact with lamin A/C and B1 [21,24]. Lamins are not only located at the nuclear membrane periphery but are also found within the nucleus [41,42]. Indeed, we showed that LAP1B co-localizes with lamin B1 at the nuclear envelope and also in specific intranuclear areas (Figure 6). Originally, nuclear lamins were proposed to have a role in supporting the nuclear envelope and binding to chromatin. However, recent reports suggest many other roles for nuclear lamins, namely in DNA replication, transcription, mitosis, apoptosis and cell differentiation [43]. Potentially, and in a similar fashion, LAP1B may be involved in other nuclear functions. LAP1B is known to be phosphorylated during both interphase and mitosis and several phosphorylated residues have been identified [36-38]. However, the involvement of specific kinases and/or phosphatases had not hitherto been documented. Since we validated the interaction between LAP1B and PP1 it is reasonable to deduce that LAP1B may indeed be a substrate for PP1. When PP1 is inhibited with OA a slight shift in the LAP1B migration is observed, consistent with an increase in its protein phosphorylation state (Figure 8). Additionally, upon adding purified PP1γ1 protein to LAP1B immunoprecipitates, from cells previously incubated with 500 nM OA, an increase in the migration of LAP1B was detected, indicating that *in vitro* PP1 dephosphorylates LAP1B.

Many PP1 regulators identified thus far are located in the nucleus and are involved in diverse cellular functions, such as, cell cycle regulation, splicing and transcription [4,5,20]. However, the only known PP1 regulator located specifically at the nuclear membrane is AKAP-149. AKAP149 is a component of the endoplasmic reticulum/nuclear system but the discovery that it interacts with lamins A/C and B [44], suggests that AKAP149 is associated with both the outer and inner nuclear membranes. AKAP-149 recruits PP1 to the nuclear envelope upon nuclear envelope assembly *in vitro* and promotes lamin B dephosphorylation and polymerization [45,46]. Indeed, PP1 may mediate nuclear lamina reassembly, in part by dephosphorylation of lamin B at the end of mitosis [47]. Following nuclear envelope disassembly at mitosis, LAP1B and lamin B have similar localization and both reassemble around the nuclear envelope during telophase [48].

In conclusion, we have identified a novel PP1 regulatory protein, LAP1B. Both proteins co-localize in the close proximity of the nuclear envelope. LAP1B is mainly localized to the nuclear envelope whereas PP1 is rich in the nucleus. Co-localization of the LAP1B:PP1 to the nuclear envelope is indicative of specific protein recruitment for signaling events, and this will be addressed in the future. This interaction occurs through the REVRF motif, located in the nucleoplasmic domain of LAP1B. Further we also determined that LAP1B is a substrate for PP1 and PP1 in turn will dephosphorylate LAP1B.

## Supporting Information

Table S1 **Oligonucleotides used to generate LAP1B deletion mutants by PCR.**
(DOCX)

Table S2 **Summary of LAP1B clones isolated from the yeast-two hybrid (YTH) screens.**
(DOCX)
