# Supplementary material for: The Nuclear Envelope Protein, LAP1B, Is a Novel Protein Phosphatase 1 Substrate
Source: PLoS One. 2013 Oct 7;8(10):e76788. doi: 10.1371/journal.pone.0076788 (PMC3792071; doi:10.1371/journal.pone.0076788)
Supplement: Table S1 — Oligonucleotides used to generate LAP1B deletion mutants by PCR. (DOCX) [file pone.0076788.s001.docx]

**Table S1.** Oligonucleotides used to generate LAP1B deletion mutants by PCR.

|  | **Plasmid name** | **Oligonucleotide sequence (5’ – 3’)** | |
| --- | --- | --- | --- |
|  |  | **FW** | **RV** |
| **pACT2 vector**  **constructs** | LAP1B-BM1 | GGAATTCATATGGCGGGCGACGGG | CCGCTCGAGTTAGACACTGGTGGCTTC |
|  | LAP1B-BM2 | GGAATTCATATGGACGAGCCGCCAGAA | CCGCTCGAGTTAGGCTACATCTTTGAAGGC |
|  | LAP1B-BM1/2 | GGAATTCATATGGCGGGCGACGGG | CCGCTCGAGTTAGACACTGGTGGCTTC |
|  | LAP1B-BM3 | GGAATTCATATGGCCAGATCCAGGGAT | CCTCGAGTTATAAGCAGATGCCCCT |
|  | | | |
| **pET-28c vector**  **constructs** | LAP1B-BM1/2+TM | GGAATTCATATGGCGGGCGACGGG | CCTCGAGTTAGAACTCTTGAACAG |
|  | LAP1B-BM1/2-TM | GGAATTCATATGGCGGGCGACGGG | CCTCGAGTTACCGGTTCCTCTTGAC |
|  | LAP1B-BM3+TM | GGAATTCATATGGTCAAGAGGACGG | CCTCGAGTTATAAGCAGATGCCCCT |
|  | LAP1B-BM3-TM | GGAATTCATATGGCTGTTCAAGTTC | CCTCGAGTTATAAGCAGATGCCCCT |
|  | LAP1B (ΔA185) | CATACAAGAGGCTCCAGTGAGTGAAGATCTTG | CAAGATCTTCACTCACTGGAGCCTCTTGTATG |
