# Supplementary material for: The Nuclear Envelope Protein, LAP1B, Is a Novel Protein Phosphatase 1 Substrate
Source: PLoS One. 2013 Oct 7;8(10):e76788. doi: 10.1371/journal.pone.0076788 (PMC3792071; doi:10.1371/journal.pone.0076788)
Supplement: Table S2 — Summary of LAP1B clones isolated from the yeast-two hybrid (YTH) screens. (DOCX) [file pone.0076788.s002.docx]

Table S2. Summary of LAP1B clones isolated from the yeast-two hybrid (YTH) screens.

| **Bait** | **Nº of screened clones** | **Nº of positive clones** | **Nº of LAP1B clones** | **YTH clone ID** | **Insert (Kb)** | **Start position** |
| --- | --- | --- | --- | --- | --- | --- |
| **PP1α** | 2 x 10^7^ | 298 | 14 | 12 | 2.0 | 384 |
|  |  |  |  | 31 | 1.75 | 382 |
|  |  |  |  | 36 | 2.25 | 268 |
|  |  |  |  | 45 | 2.15 | 382 |
|  |  |  |  | 50 | 2.75 | 274 |
|  |  |  |  | 61 | 3.85 | 265 |
|  |  |  |  | 76 | 3.5 | 262 |
|  |  |  |  | 96 | 2.2 | 383 |
|  |  |  |  | 184 | 4.3 | 265 |
|  |  |  |  | 192 | 3.75 | 274 |
|  |  |  |  | 261 | 4.0 | 265 |
|  |  |  |  | 262 | 2.1 | 382 |
|  |  |  |  | 271 | 2.2 | 382 |
|  |  |  |  | 273 | 3.75 | 365 |
| **PP1γ1** | 1 x 10^6^ | 241 | 4 | 120 | 2.4 | 261 |
|  |  |  |  | 124 | 1.5 | 266 |
|  |  |  |  | 135 | 2.0 | 382 |
|  |  |  |  | 164 | 1.6 | 268 |
| **PP1γ2** | 6.6 x 10^5^ | 228 | 2 | 124 | 1.8 | 262 |
|  |  |  |  | 164 | 0.3 | 270 |

Clone 135 (interaction with PP1γ1) was selected for further study. The start position indicated is relative to the GenBank sequence NM_001267578.
